# A Collaborative Location Based Travel Recommendation System through Enhanced Rating Prediction for the Group of Users

**DOI:** 10.1155/2016/1291358

**Published:** 2016-03-16

**Authors:** Logesh Ravi, Subramaniyaswamy Vairavasundaram

**Affiliations:** School of Computing, SASTRA University, Thanjavur, Tamil Nadu 613401, India

## Abstract

Rapid growth of web and its applications has created a colossal importance for recommender systems. Being applied in various domains, recommender systems were designed to generate suggestions such as items or services based on user interests. Basically, recommender systems experience many issues which reflects dwindled effectiveness. Integrating powerful data management techniques to recommender systems can address such issues and the recommendations quality can be increased significantly. Recent research on recommender systems reveals an idea of utilizing social network data to enhance traditional recommender system with better prediction and improved accuracy. This paper expresses views on social network data based recommender systems by considering usage of various recommendation algorithms, functionalities of systems, different types of interfaces, filtering techniques, and artificial intelligence techniques. After examining the depths of objectives, methodologies, and data sources of the existing models, the paper helps anyone interested in the development of travel recommendation systems and facilitates future research direction. We have also proposed a location recommendation system based on social pertinent trust walker (SPTW) and compared the results with the existing baseline random walk models. Later, we have enhanced the SPTW model for group of users recommendations. The results obtained from the experiments have been presented.

## 1. Introduction

The enormous growth of web and its user base has become source for large amount of information available online. This information may be helpful for users, to suggest items or services as per their preferences. Recommender system plays the role of generating suggestions by collecting user information such as preferences, interests, and locations. The research on recommender systems gained importance after the emergence of collaborative filtering [1, 2]. Various researches led to the implementation of recommender systems to different domains such as e-commerce [3], advertising [4], and tourism [5]. Generating suggestions according to user preferences is a complex task for recommender systems. Semantic web technologies help recommender systems to resolve those tasks easily [6]. Recommender system uses information from many sources to make predictions and to suggest an item for a user. Factors such as novelty, stability, and accuracy are balanced in the generated recommendations. Filtering mechanisms play an important role in the recommendation process [7]. The most commonly used filtering techniques are collaborative filtering, content-based filtering, knowledge-based filtering, and social filtering [8]. Already many researches had contributed to the development of various recommender systems such as movie [9, 10], music [11, 12], books [13], e-commerce [14–16], e-learning [17, 18], web search [19], and tourism [20].

The main aim of using personalization techniques is to generate customized recommendation according to the user preferences and interests [21]. The recommender system has an objective to filter unwanted information and to provide specific results for the particular user [22]. In the travel recommender systems [20], proposed model learns the user preferences and generates places of attractions according to the user interests. This paper focuses on the recommender systems and their application in tourism. To make this paper useful to all, including new readers of recommender systems, it covers topics from evolution to applications along with the challenges in it. Since more research is required to improve the effectiveness and efficiency of recommender systems, this paper will be more useful to the upcoming researchers to develop a user specific recommender system.

This paper contributes clear review of recommender systems published in scientific journals and conferences with a special focus on travel recommender systems. These systems are analyzed through the recommendation mechanism, interface, data source, and functionalities used. The paper also provides some guidelines to develop efficient, user specific travel recommender systems.

As a description about the methodology of collection and organization of articles for the analysis on the travel recommendation problem, a starting study was performed to focus on the most illustrative subjects and terms in the recommender system field. Initially, 105 recommender system papers were chosen from various journals and conference publications, with a higher need for current and frequently referred to articles. Next, we extricated from these papers the most considerable terms. We gave the most accentuation to decisive words, less accentuation to titles, and, at last, the minimum accentuation to modified works.

The paper gives a clear view on recommender systems and explains the various problems in the traditional recommender systems. Then, we have explained the development of travel recommender systems along with the techniques and interface types. Later, the location based social network was introduced and its needs and issues were explained in detail. As for the part of proposed model, we have explained in depth about SPTW (social pertinent trust walker) model for category of location recommendations. For the enhancement of the proposed location recommendation model, we have introduced SPTW based group recommendation model (SPTW-GRM) for group of users. The proposed models have proved their efficiency and accuracy through evaluation metrics.

The paper is organized as follows. The next section describes the significance of recommender systems.  Section 3 explains briefly about travel recommender systems and Section 4 briefs the use of AI techniques in tourism recommender systems. Then, Section 5 discusses in brief location based social networks and Section 6 portrays the proposed social pertinent trust walker (SPTW) algorithm.  Section 7 describes the proposed SPTW based group recommendation model (SPTW-GRM) and Section 8 illustrates evaluation of the proposed model and discussion on the results. Finally, the paper concludes with the analysis of surveyed systems and Section 9 also indicates the new areas to be focused on in the area of travel recommender system in future.

## 2. Significance of Recommender Systems

**Figure 1:**
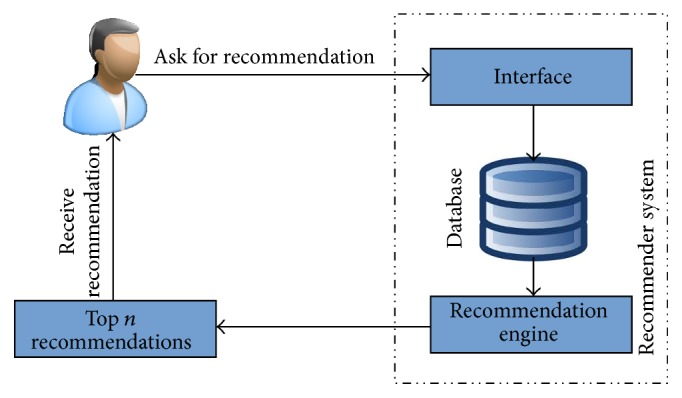
Traditional recommender system.

**Figure 2:**
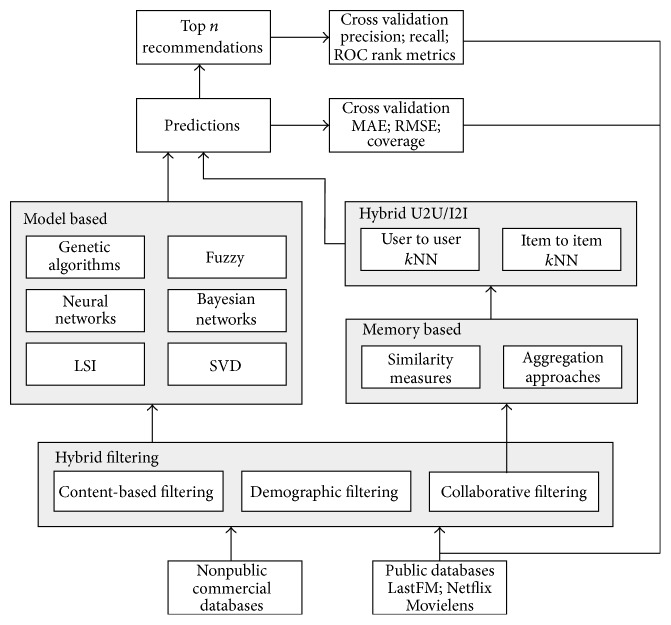
Conventional work flow model in a recommender system.

Recommender systems (RSs) were generally defined as expert systems which are used to recommend products or services to the users.  Figure 1 portrays the working of a traditional recommender system. Various factors influence the interests of users to make a decision regarding the recommended products or services. By using community-contributed data, such as blogs, social networks, Geographic Positioning Systems (GPS) logs, and geotagged photos, recommender systems tend to help the users by generating personalized recommendations, which will be more useful for the users in their decision making process.  Figure 2 describes the conventional work flow model in a recommender system.

### 2.1. Foundations of Recommender Systems

Traditionally, recommender systems are based on their building blocks such as algorithms, filtering methodologies, taxonomies, and databases. When the recommender systems have only small amount of data for generating suggestions, collaborative models face issues with them. Such problem is called cold start problem and it is described below. Then, the section describes *k*NN algorithm, which is mostly used by collaborative filtering based recommender systems. The usage of similarity and differences between users' interests is mostly used by many recommendation models. Finally, by comparing the users or items, different similarity measures were described.

#### 2.1.1. Fundamentals

In the recommender systems, process of generating recommendations depends on various factors, such as the following:

(i) available user data in the database (such as user information, interests, ratings, locations, and social relationships);
(ii) filtering mechanism/algorithm used (like, content-based, hybrid, collaborative, etc.);
(iii) techniques used to enhance the results (such as Bayesian networks, singular value decomposition, and fuzzy models);
(iv) sparsity level and scalability of database;
(v) system performance (such as memory and time consumption);
(vi) considered objectives of the system (such as top recommendations and predictions);
(vii) quality and its metrics used for the result and analysis (such as precision, recall, *F*-measure, and novelty).

Public databases are used in the research of recommender systems to develop new methods, techniques, and algorithms. Delicious and last.fm are the most popular databases used in the development of recommender systems.

#### 2.1.2. Cold Start Problem

The problem of generating nonreliable recommendations due to lack of initial ratings is known as cold start problem [22, 23]. New user, new item, and new community are the three types of cold start problems. During recommender systems' operations, new user problem [24, 25] is a great difficulty in producing personalized recommendations. Since there are no user ratings provided by these new users, memory-based content filtering cannot help in the recommendations. New users may reject unreliable, nonpersonalized recommendations and the recommendation services too. Adding additional information to the new user database, such as preferences, tackles the new user problem. Similarly, new item problem [26, 27] arises due to addition of new items in the recommender systems. Since there is no initial rating for these new items added to the recommender systems, it gets unnoticed by most of the users and large group of users may be unaware of such items. Developing a set of motivational users to rate the new items will help in solving new items problem. New community problem [28] occurs during the initialization of recommender systems due to insufficient ratings. Collaborative filtering based recommendations and encouraging users to rate items can easily solve the new community problem.

#### 2.1.3. The *k* Nearest Neighbors Recommendation Algorithm

In most of the recommendation processes, using collaborative filtering, the reference algorithm used is *k* Nearest Neighbors (*k*NN) recommendation algorithm. *k*NN recommendation algorithm is simple and reasonably produces accurate results. The drawback of the *k*NN recommendation algorithm is low scalability [29] and it is vulnerable to the high level of sparsity [29, 30] in recommender systems databases. The *k*NN algorithm focuses on similarity measures and generally similarity computation is carried out between user to user [31], item to item [32, 33], and user to item [34]. Using similarity measures, similar users are assigned as neighbors to the user and items recommendation is predicted for the user. Then, from the top-*n* recommendations, *n* items are chosen to satisfy the particular active user.

#### 2.1.4. Similarity Measures

The similarity between users or items can be determined by similarity metric or similarity measure. The most commonly used traditional metrics are cosine (COS), adjusted cosine (ACOS), Pearson correlation (CORR), constrained correlation (CCOR), Euclidean (EUC), and Mean Squared Differences (MSD) [22, 35]. There are also few new metrics used, such as Jaccard Mean of the Squared Differences (JMSD) [36] calculated by using nonnumerical information, Singularity (SING) [37] premeditated by utilizing the information in the user votes, GEN [31] used as a similarity measure in the recommender system which uses the genetic algorithms, NCS [37] used as a similarity measure in the recommender system which uses neural learning, TRUST [38] which utilizes the reputation of users' ratings on items, and UERROR [39] which predicts the initial actual ratings of the user and determines the predictions errors too.

## 3. Travel Recommender Systems

The large amount of user information available is exploited by the travel recommender systems to provide suggestions to the user in the effective manner [40]. The tourism/travel recommender system employs artificial intelligence techniques to generate personalized recommendations to the user. This section depicts the applications of the recommender systems in the field of e-Tourism.

### 3.1. Interface

**Figure 3:**
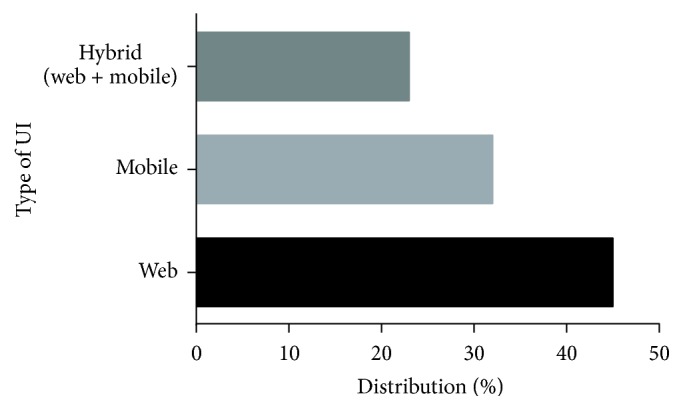
Statistics of interfaces used in the existing systems.

This section presents an overview of different interfaces prevalent in recommender systems today. Interfaces are web oriented or mobile device based; some are designed with compatibility of both web and mobile device in mind. Classification of recommenders is based on two major categories (namely, web based and mobile based).  Figure 3 describes the statistics of interface used in the existing systems in percentage. Users were found to give high preference to web based interface since it provides ease of use from any computer without the need of download, installation, and configuration procedures. But due to the rapid increase in the number of mobile devices, especially Smartphones, most web based recommender systems also have a mobile device based counterpart.

Some systems are desktop specific and they do not support web oriented or mobile device based interfaces (e.g., [41]). It has to be noted that these systems are easier to develop and implement. But they are not popular among the tourists, since downloading, installing, and configuring are preliminary parts of their usage. Tourists tend to prefer very simplistic approach to get their recommendations.

#### 3.1.1. Web Based Recommenders

Web based interface is the most prevalent among the interfaces used for e-Tourism recommender systems. It is highly user friendly with various interaction options for the users to get information from maps, images, and videos. The possibility of mouse usage allows the user to explore maps by zoom, select, and drag-drop options. Web based systems are thus suitable for the planning stages of the tour. The design of these systems limits the usage to computer systems, the unavailability of which is a concern that a tourist has to keep in mind. The availability of large desktop screen to provide easily retrievable information is a key advantage of these systems. We now provide exploited features of this type of recommender systems. In Venkataiaha et al. [42], a comparative study has been made between the so-called discrete and continuous systems. In discrete systems, the screen is utilized to a high extent providing the users with the needed information, whereas the continuous system combines the associated media, text, photographs, and video contents into a single video clip, thereby reducing the efforts required by the user to understand the contents provided.

The proposed work of Lee et al. [43] is the first approach that incorporated Google Maps Services for the web interface allowing the plotting of paths on map, guiding the user through the personalized route to the selected locations and food places at the Tainan City. City Trip Planner [44], e-Tourism [45], and Otium [46] are some of the web based systems in which a map is marked with the scheduled locations to be visited for a single day. EnoSigTur [47] also uses the same approach in addition to which the user's preferences and sociodemographic information is obtained, after which the recommendations are made.

An Avatar-based interactive approach that allows the users to provide their requirements has been implemented by the VIBE virtual spa advisor [48]. This allows dynamic addition of new attributes to the catalogue which in turn automates the changes in recommendation, preference elicitation, and also the web interface process. It also includes section where domain experts can manipulate the conversational and recommendation procedures.

An ontology based recommender system by Wang et al. [49] uses semantic web technologies coupled with Web 2.0 services which integrates the different information from the user during the travel. It is an Ajax web based application developed on Ruby on Rails which includes other third-party services such as Yahoo weather, Google Map, and Wiki Travel.

#### 3.1.2. Mobile Recommendations

Mobile based recommendations system is increasing in the recent years due to the availability of mobile devices that support Internet facility and the Smartphones. Mobile recommender systems are designed in such a way that only the relevant and essential information is provided to the user since the Internet connection would be slow and also the amount of information that can be effectively displayed is lesser than that of a standard web page. It has to be noted that, apart from the modern Smartphones which provide easier touch based interaction, in the mobile based systems it is difficult to execute actions such as scrolling compared to standard web page. The advantage of the mobile based systems is that it can be used in any place where Internet connection is available; this can be during the travel which allows acquiring of online information that can be used to further enhance the recommendations. The GPS which is a part of most of the mobile devices is also used to locate the user which in turn helps in providing relevant recommendations.

Yu and Ping Chang [50] report a system that can be considered the first to implement mobile system based approach which was designed for PDAs. Recommendations based on the user-location and planning suggestions for tour were the services provided by the system which took into account user's location, time of access, and preferences. The different services provided by the system, the user preferences setting, the tour plan recommendations, and the usage of Google Maps can be seen.

MTRS [51] is also a PDA based approach which considers the problem of Internet connectivity. The Internet connectivity is a problem for the tourist either because of the rural area or because of the high cost during roaming. Proximity detection along with a method to update the content with minimal cost was proposed which use wireless sensor networks that would be implemented in small or medium scale range. The infrastructure would allow placing higher weight to the ratings of users from fixed connection facilities compared to users who are away from the visited locations and rate using Internet.

MapMobyRek [52] is another mobile based system that takes advantages of the interface by using maps and lists to provide the recommendations effectively. Comparison of places and items based on their characteristics was facilitated by side-by-side display which helped us to decide among recommendations. Another product that serves as a tourist guide by describing the recommended locations when the user is near them is GeOasis [53]. It utilizes the device GPS to locate the tourist and the travelling speed so that the estimation of time can be done to prepare the explanations. The user is provided with two-way interaction option, using voice recognition or the tactile interface.

Nowadays, the newest technologies that include Android and iPhone platforms are targeted for the development of mobile tourism recommenders as there is a continuous increase of its user base. We now show some of the popular systems that make use of these platforms. MoreTourism [54] is system that is based on Android which makes use of videos, images, mashups, geolocation, and other available features to aid the user in getting information. The EnoSigTur [47] is also a system that uses the Android platform for place recommendation, route aiding for trips, and description of place of interest.

The user is able to provide the push information in accordance with their context in the LiveCities recommender system [55] which makes use of the notification service of the Android platform. The push information may be text, video, audio, or HTML. STS system [56] allows the user to provide accurate information of interests, opinions, and descriptions of the visited places and turns it into a powerful application based on the Android platform. This is achieved through a user friendly and intuitive design.

The GUIDEME [57] is a recent system which can be noted for its design and implementation, since it has a compatible app for both phones and tablet devices. It is designed for the iOS platform with adaptive features that would allow adjusting with respect to the screen sizes thereby making it usable in both the iPhone and the iPad devices. Another iOS platform based system is REJA [58].

### 3.2. Functionalities

We describe the general function features of the reviewed tourism recommender systems for the approaches in four groups which are based on the recommendation of suggestions and the content of tourist package provided, suggestion of attractions of a particular location, design of long trips with schedules, and social media capabilities. In the subsections that follow we comment further about these capabilities with relevant examples.  Table 1 summarizes the comparison of travel recommender system based on its interface and functionalities.

#### 3.2.1. Travel Destination and Tourist Packs

User's preference is taken into consideration in some of the systems to provide the recommendation so that it suits the user. PersonalTour [59], Itchy Feet [60], and MyTravelPal [61] are of this type. PersonalTour is a recommender system that is used by travel agencies in order to find suitable travel packages in accordance with the customer preference. The recommendation is made in the form of a list of suggestions. Then the rating of each travel service from each item can be done by the customer.

Itchy Feet allows the purchase of services that would book trips, assistance, and other services along with the recommendation of the locations. It uses both the internal database and external data sources when a user makes a search request. Its interface allows the user to select from the result items shown such as a list of flights or hotels.

MyTravelPal [61], in accordance with the affinity to user areas of interest, is recommended first graphically. When an area is chosen, further recommendations of tourist spots and services are listed based on the preferences of the user.

#### 3.2.2. Ranked List of Suggested Attractions

Recommender systems for tourism generally provide suggestions only after acquiring the information such as the destination and cost beforehand from the user. This leads to listing out many attractions, temporal events, and other places of interest. Hence, these systems are more complex as the system classifies and ranks relevant suggestions from a huge database of available information. The suggested list of attractions helps the user to spot places of interests in an efficient manner and supports him in discovering more about the locations. Static database is commonly used for storing the elements for recommendation. Some systems make use of the web by extracting information automatically so that updated recommendation is ensured (e.g., Otium [46]).

Some recommender systems match the preferences of the user, check the past travel history for locations, and also compare the positively reviewed locations of other users to provide a suggestion list. This is achieved by the usage of mechanisms to compare various preferences and similarities between various user profile and streaming data. Contextual factors such as user's current location may also be considered to select the recommendations [58]. Justification capability for the list of suggestions is also provided by some of the systems (e.g., [48]).

Automated detection of user's indoors or outdoors presence is made in a more complex system SMARTMUSEUM [74], which utilizes the user-location information for this purpose. When the user is outdoors, the conventional map representation is provided, whereas when the user is indoors, suitable listing of objects based on the preferences of the user is given.

#### 3.2.3. Planning a Route

Apart from providing the list of spots and locations that is relevant to the user preferences, there are some systems that guide the tourist in preparing a route plan along several places.

CT Planner [41, 71] refines the tour plans offered based on the preferences and requests of the users while selecting the plans. The user's choice of factors such as walking speed and duration is considered to plan the route. A radar chart and a cartoon character are parts of the interface that helps to navigate providing better interactivity.

In some of the systems, the users can build up or manipulate an initial plan recommended which would include activities and locations. The user can reorder the plan and route, add more activities, and schedule them according to their choice. The initial plan is designed by considering the timing of various attractions, distances to be covered in between, and the expected visit duration. Examples of this type of recommender system include EnoSigTur [47], City Trip Planner [62], Smart City [66], Otium [46], and e-Tourism [45]. Vansteenwegen and Souffriau [77] discuss in detail the functionalities of such trip planners. SAMAP [78] and PaTac [79] are some of the advanced systems that generate recommendations, by calculating the possibilities of activities with respect to different transporting facilities; that is, mode of transport, car, bike, walk, or public transport, is taken into account.

Geographical Information Systems (GIS) are incorporated by some of these systems for the management of geographical data that has to be associated with the recommended locations and activities. According to [80], spatial data in large amount is unfeasible for usage and can be maintained computationally for utilizing in planning procedures. Hence, the locations, distances, and driving directions are obtained from geospatial web service technologies already existing along with ESRI ArcWeb Service. Continuous calculation of user's position and speed is made by GeOasis [53] to estimate the time required to reach a location so that planning can be made in real time. Prediction is the key aspect of the system which determines whether the user would be on the road, in a city, or near a city. The planning algorithm will consider only the nearest places if the users are in a city already, assuming they are closer without counting the speed or route. The attractions in a city are considered if the user is near them. The algorithm gets complex due to temporal constraints when the user is on the move but far from a city. The plan is computed by the client application not the server as constant checking of GPS to determine the location is needed. Google Maps acts as an external resource to compute the routes.

Retrieval of the complete schedule along with the routes is possible by the user once it is completed. Retrieval methods differ among systems, such as EnoSigTur [47] that supports the download in PDF format that contains a map which is georeferenced along with explanations in detail. City Trip Planner [44] and Otium [46] support the downloading of the route map and details to a mobile phone.

#### 3.2.4. Social Aspects

Social functionalities have been focused on by some of the projects (such as [44, 57, 69, 79]), thus allowing the user to interact and share material such as evaluation, comments, or pictures with other tourists. This aspect promotes the usage of recommender when a user is in a particular location or activity. Some systems such as Itchy Feet [60] and MoreTourism [54] allow the user to organize events or activities with similar tourists apart from interacting and commenting. e-Tourism [65] can be made to consider the preferences of an entire group of visitors and suggest activities or route plans.

iTravel [76] allows peer-to-peer communications to share ratings and reviews of attractions. The navigation map shows the positions of users nearby along with the route and attractions. The system shows the green pins and blue pins which reflect the suggested attractions and nearby users, respectively.

Alchemy API is used by the VISIT system [73] in order to apply sentiment analysis methods so that Twitter and Facebook updates of a given attraction are analyzed to determine whether the users are providing positive or negative emotional comments regarding it. This information is conveyed to the users by the display of green and red colors in the interface indicating the most loved locations of the day by the users and those that are not.

The three main objectives for the usage of social information are (i) to enhance the prediction in terms of accuracy, (ii) to develop or design new hybrid recommender systems, and (iii) to determine the relating features between various processes and the social information.

When social information is included in the recommendation system, the item can be labeled by the users. Folksonomies are information spaces consisting of sets of triples that specify a user, an item, and a tag [81–83].

## 4. Use of AI Techniques in Tourism Recommender Systems

Now, we brief the most prevalent AI methods that have been exploited in recommender systems for tourism in the recent years.  Table 2 is a comparison of AI techniques used by travel recommender systems in the articles reviewed for this paper.

### 4.1. Multiagent Systems

Agents obtain information intelligently from the environment in which they act upon accomplishing the task or goals assigned. These software programs are proactive and automated. Optimal solutions to problems are obtained by multiagent systems where a set of agents operate by coordinating and cooperating among themselves in terms of resource and information sharing [88].

The Turist@ [67] implements a system based on agent in which suggestions for cultural activities are generated. An agent is assigned each of the concerned activities to maintain a database of events related and its availability in the location. Museum are each assigned an agent and hence are exempted. A user agent provides an interface for interaction graphically. A broker agent interfaces the communication of information in between the user agents and the agents assigned to each of the cultural activities. The user is provided with the ability to interact in order to query, search, or evaluate recommendations. The recommender agent serves as the main component of the Turist@ system by maintaining the tourist's user profiles. Automatic-dynamic tuning of the knowledge base is done which is obtained from the tourists by elicitation during the initial usage. This initial knowledge comprises the interests and cultural activity preferences of the users. Analysis and querying process are carried out in this initial knowledge in order to refine them. Proactive suggestions are possible since the current user-location can be used to determine the activities that match the user preference. The system incorporates both CL and CB suggestion methods.

In Ceccaroni et al. [79], profile obtained initially from the user is modified by the analysis of both explicit evaluations and implicit activity actions. A profile management agent apart from initializing the profile by classifying the users into stereotyped groups performs its modification based on tourist feedbacks. This work proposes having an information service agent in order to query the databases for tourist information along with a personalization agent that implements the CB methods to provide suggestion by selecting item based on the user profile and data.

The PersonalTour [59] implements travel agents that are assigned to specific feature such as hotels, attractions, or flights. Upon the arrival of a new user, the preferences and interests are elicited which then are processed together with the implemented agents to derive a suitable package for travel. Evaluation of the package content and segments is possible by the user, which in turn is regarded as an implicit feedback that helps in improving the performance of the agent used and the suggestions provided by the system in general.

Castillo et al. [78], Lee et al. [43], and Sebastia et al. [63] studies are some of the examples in which the system is built by the combination of agents that correspond to different operational components such as the user interface, preference and interest elicitation module, analysis and matching module, and route planning and generation module. In order to keep the system simple, communication and coordination among the agents are kept minimal, which is achieved by the sequential activation of the arranged agents. This limits the agents' capabilities in terms of being concurrent, distributed, and coordinated.

### 4.2. Optimization Techniques

Complicated plans and schedules have to be generated by the recommendation systems, which leads to a situation in which solutions to related problems that are NP complete have to be optimally approximated. Computational complexity is often considered as a trade-off in this regard; hence, suboptimal methods are also considered in some cases.

Ant colony optimization is used by Lee et al. [43], in the design of a route recommendation system that incorporated agents. Optimal coverage of the attractions along a route is obtained here by approximating a solution of travelling salesman problem by an autonomous entity set representing the ants that globally and indirectly communicate through pheromone mediation. Genetic algorithm for the plan construction of a city tour is implemented in CT-Planner4 [71]. It is an iterative approach that considers the possible plans as the population with the user utility as the evaluation function. The iterations are made after the mutation and crossover of the determined best population. The iterations lead to the selection of the best plan evaluated with respect to the user preference. In VISIT system [73] adaptive suggestions based on the user context with respect to factors such as social media sentiment, preference, weather, time, and location are proposed. Artificial neural network is suggested for the evaluation of the relevance between each context factor used and the user profile.

Vansteenwegen et al. [44] and Garcia et al. [69] are some of the works that use heuristic based approaches to develop the travel routes. Another example is the City Planner system. Iterated local search can be also used in this kind of approaches by which generations of solution sequences with respect to local search can be iterated. A different solution to a route plan can be directed efficiently by the addition of heuristic after which the optimal solution can be considered as the initial solution with respect to the local search. Iteration can thus be continued till a threshold criterion is achieved. In Souffriau et al.'s study [89] of Greedy Randomized Adaptive Search techniques, query iterations generate a set of possible locations or attractions that were generated from the chosen start and end locations of the tour. The set thus obtained is filtered with respect to the heuristic threshold value established initially and a random selection is made on the remaining items.

A^*∗*^ heuristic and hierarchical temporal planning were exploited in the SAMAP system [78]. Apart from these methods, recommender system for tourism is widely incorporated with ad hoc planning methods in order to generate the routes and plans that are personalized for a specific user. Also classical AI methods which are independent of domain are also applied by some systems.

### 4.3. Automatic Clustering

CL methods in tour suggestion systems are generally used to group the users based on the common attributes and features shared. Here, it is assumed that it is appropriate to suggest to all other members of a group an activity or location that has been rated positively by a user of the group. Information based on user demographics, user preference, and rating history is used to determine these groups based on similarity by applying automated clustering techniques based on AI. We now further consider alternatives of this approach.

*k* Nearest Neighbors method is used to determine in which group a new user has to be added [90], which involves calculating the *k* past users who are similar to the new one such as in [58, 91]. Once the new user has been allotted into one of the groups, the suggestions can be given with respect to the interests and preferences of the rest of the group members. Domain ontology is analyzed to determine the similarity index between the users of a group in SAMAP [78]. One problem that has to be given attention here is scalability.

Another method that is generally used to group similar users is the *k*-means algorithm such as in Gavalas and Kenteris [51] and Lucas et al.'s [72]. Here, *k* is the number of clusters desired with respect to which the initial seeds are determined independent of the application. This is followed by iteration of objects, sorting based on the calculation of the nearest cluster, and recalculation of the cluster prototypes. Convergence of solution is achieved when repeated iterations place the objects under the same cluster. Application of *k*-means for determining an initial set of tour segments, classes of similar users based on demography, and classification with respect to explicit feedbacks is done in Moreno et al. [87]. 100 generic initial segments as types of users were determined based on historical data that comprised 30,000 questionnaires. Each segment is related to a prototype and level of preference with respect to each type of activity. Basic information is elicited from a new user by means of explicit feedback forms which can be sufficiently used to place the user in one of the groups and provide initial suggestions. The composition of travel group, budget type of accommodation, and country of origin are the demographics considered in order to classify the user. In order to combine various kinds of data, operators of aggregation such as LSP [92] and OWA [93] are used.

In Fenza et al. [85], a variation of *k*-means in terms of uncertainty, the fuzzy *c*-means, is proposed, by which the object sets can be partitioned into clusters in such a way that every object membership degree lies between 0 and 1. And for all clusters the addition of membership degree is 1. The algorithm handles both the point of interest (poi) and the users. Once the POIs and the user clusters are defined rules are derived, characterizing them so that the new user or POI can be placed in the best fitting cluster. The association rules can also be built in such a way that they capture the relation between the POI and user clusters along with other pieces of information that are contextual. The rules obtained can then be used to calculate the activities and their types that can be suggested to a user. Similar methods can be found in the recommender system PSIS (Personalized Sightseeing Information System) [72].

CL filtering methods that use class definition of grouped users are implemented in Turist@ [67]. Whenever the system gets an addition of 10 new users, clustering is redone to update the classes. In ClusDM [94], user interests and preference along with the corresponding demographic data are used to build a class hierarchy. Desired number of classes can be obtained by segmenting the tree thus obtained with respect to different levels.

In SPETA system [95] classification by the usage of Support Vector Machines (SVMs) is proposed. For this, storage of user preference of various activities and its corresponding characteristics in the form of vectors is made, upon which the SVMs can be used to determine the suitable and most appropriate suggestions by analyzing the distance between the item sets and the user preferences.

### 4.4. Management of Uncertainty

Determination of suitable suggestions to the users is difficult as the calculation of the relation between the user demographics, preferences, and the available POIs of a location involves complex methods. This leads to uncertainty in the system which can be addressed by AI method that involves approximate reasoning techniques that can determine and reason these uncertain relations.

Bayesian networks are one such possibility, in which acyclic graph with representation of causality relations or internodal influences can be made in terms of the edges. Probability analysis is used to determine the possible or the most appropriate parent for a node in case of its absence. A probability table is used for this purpose. The table of conditional probability consists of 2*n* nodes for a node that has *n* parents. This table indicates the chance of occurrence based on the parent nodes presence or absence. Hsu et al. [86] present a simple method that involves the Bayesian networks to determine the probability of POI to be preferred by a user by considering various attributes such as nationality, age, income, occupation, and travel purpose. The proposed network considers that the probability that a user is likely to prefer an activity or location is influenced by factors such as age, personality, and occupation. Activities or events are not specified in the used network.

Fuzzy logic is also widely used to handle the uncertainty in various systems. Linguistic variable values are processed by this method by operation on a series of values. The variables belong to a fuzzy set in which the corresponding values are mapped to a fuzzy membership function that results in values between 0 and 1. Thus, a generalization of standard logic is achieved. The fuzzy logic can be used to represent the user preferences and demographic and the possible item and activities list and map the possibility of a user choosing an item [43, 95]. The measure of how similar a user group or a particular user is with respect to other users or groups can be determined using the fuzzy logic analysis [72]. Also the representation of contextual aspects pertaining to travel route or path can be done [73].

In some of the recommendation systems, fuzzy component is avoided by the usage of rule based methods. CONCERT system [96] involves suggestions generation by the rules that consider the user context and preferences.

### 4.5. Knowledge Representation

Representation of the domain knowledge in tourism recommendation systems requires methods that would be efficient and effective in inference mechanisms such as in any knowledge or rule based system. AI techniques are found to be adequate to represent and build the knowledge base and to derive reasons from it. In particular, ontologies are widely used nowadays for the domain knowledge representation. Classes that represent the concepts and described hierarchical relations that are taxonomical and nontaxonomical are the important components of such systems [97, 98]. Apart from these axioms and specific objects, other components are usually considered [99].

In some of the recommender systems for tourism, ontology based formalization of the domain knowledge is made. Cultural activity suggestions are made by considering generic ontologies in which information regarding various aspects is stored, such as Wang et al. [49], in which the formation with respect to aspects such as restaurants, shopping, transport, accommodation, and culture comprises the generic travel ontology. The user ontology modeling considers the user preferences and demographics. In GeOasis [53], SAMAP [78], and SMARTMUSEUM [74], there is inclusion of ontologies for modeling various kinds of user activities and items and for semantic reasoning. Ontology was again used for similarity measuring and deduction of similar items or groups which along with CL filtering methods were used to generate the suggestions.

In e-Tourism [45, 63, 65], methods based on ontology are used extensively. Domain ontologies which have the details of various activities and events of a city are used. Ontology is used in SigTur that comprises concepts greater than 200, hieratically arranged in 5 levels.

In SigTur, the storage of user preference is made in each of the ontology nodes along with the confidence level of the considered preference. The initialization with respect to these ontology references is elicited from the new user with the help of a small questionnaire. The system is updated by the transmission and spreading of information from the children nodes to the parents when the user interacts with the system. The interaction may be a search, an addition of certain activity to a generated plan, or any usage that provides additional knowledge about the user preference. Hence, the user preferences are managed dynamically based on ontology [68]. Similarly in the e-Tourism recommender system, preference updates are made after the analysis of explicit user ratings.

An ontology set instead of integrated single ontology has been proposed in some of the systems. In PaTac [79], a separated ontology is maintained for activities and locations like entertainment, restaurants, and hotels. The work proposes the linking of ontologies based on user models based on user stereotypes and the ontologies of W3C consortium which has temporal standard and is based on geolocation. In CONCERT [96], “ContOlogy” is used which is the inclusion of 11 different ontologies that relate to attributes such as services, tourism, preferences, motivation, or activities to model an ontology network that considers all the travel related contexts.

Usually the design and development of ontologies in the recommender systems are manually built in an ad hoc manner for a particular application. In [100], ontology population is automated in order to minimize its construction cost. This is done by the analysis of electronic resources.

## 5. Location Based Social Networks

More than one number of individuals connected together with more than one type of relations (e.g., friends, family, common interests, and groups) is known as social network [101]. A real world social network service can be digitally represented. The social network not only mentions the users' network, but also enhances their activities. The activities of a user depend on their actual ideas and on sharing posts and events and making likes.

The user-location based social network data strengthens the social network activities and also this location mentioned in the social network services. Location based social network formal definition is proposed by Zheng [102].

The location based social network comprises the people's physical location in their social structure to share the information by location embedded system. The new structure is created when an individual user is connected to a location on a social network. The location of user derived from their location tagged media content and other activities (such as their photos, video, and text). The user physical location consists of individual location at current time and their location history with specific period of time. If one or more person has the same location and also similar location histories, it will not affect our social network structure. This structure also contains individual behaviors, activities, and other information.

The concept of locations based networks shows new locations and correlations in addition to the old one. From the new information, graphs build into three types of location based social network, such as location-location graphs, user-location graph, and user-user graph.

*(i) Location-Location Graph*. In this graph, users consecutively visit the edge between two locations indicating the node location of the location-location graph. The correlation between strengths of two locations is represented by edge weight.

*(ii) User-Location Graph*. Users and locations are the two types of entities in user-location graph. The visited location of the users is indicated by the edge starting from the users and ending at a location and the number of visits calculated by weight of the edge.

*(iii) User-User Graph*. Basically a node is a user and edge between two nodes represents two relations. The two relations are existing social network between two users and a new location of the users. 

Three groups of location based social networking services are geotagged-media-based services, point location based services, and trajectory-based services.

*(i) Geotagged-Media-Based*. Locations are labeled to media content of users added by geotagging services. The new content of users is passively added to the physical world and also this content in the geographic context is viewed by the users. This location based social networking service is included in website (Flickr, Panoramio, and Geo-twitter). The social network services still focus on media content because the connection between users is based on media itself.

*(ii) Point Location Based*. Some applications like foursquare and Google Latitude mainly focus on people current locations, such as hotel or park. Foursquare application is used to point out the individual with the most number of check-ins and a popular place with higher crowd ratings. The users' real time location can be discovered by social network and also this enables the social activities of the users in the real world. These real time location social activities are useful for inviting people to have dinner or go shopping with users.

*(iii) Trajectory-Based*. The point locations and the route connecting the point location are recorded by users and are called trajectory-based social networking services (such as Bikely, SportsDo, and Microsoft GeoLife). Normally, the users' experiences are represented by their tags, such as photos, media, and tips, along the trajectories and also these services are used to record users basic information, such as distance, duration, and velocity. In addition to social networking services, trajectory-based service systems also provide the when and where information of users for personalization.

### 5.1. Unique Properties of Locations

**Figure 4:**
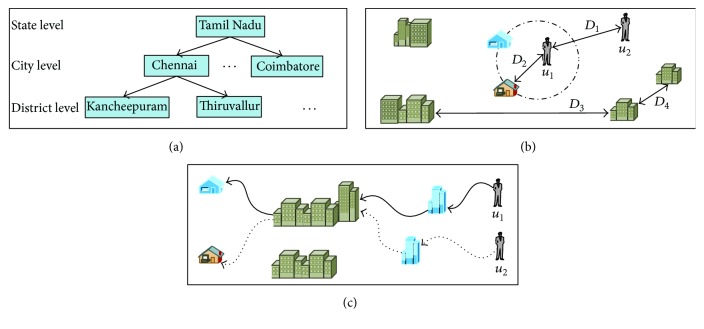
(a) Location hierarchy property. (b) Location distance property. (c) Location sequential property.

Three unique properties of location based social network are hierarchical, measurable distances, and sequential ordering properties as shown in Figure 4.

#### 5.1.1. Hierarchical Properties

Multiple scales in location span; that is, the location can be hotel or town and it depends on users' location. The different granularities of a location form a hierarchy; the locations with smaller area also connected with geographic areas. Each location has a relation with another; for example, a hotel belongs to a neighborhood, the neighborhood belongs to a town, and a town belongs to a country; location-location graphs and user-location graphs have a location granularity in different levels (Figure 4(a)). The hierarchical relationships depend on users with lower level location sharing and higher level location sharing. The lower level has a stronger connection than higher level location sharing. This is a unique property of location based social networks.

#### 5.1.2. Measurable Distances

The new geospatial distance relation has three types of location based social network that connected to physical world, different users locations with their distance, user and location distance, and distance between two users locations. The first law of geography stated that “everything is related to everything else, but near things are more related than distant things” (Figure 4(b)). Location based social networks are affected by the influences of similarity between user-user distances; for example, the most visited location of user has more preferences [103], and the users are interested in particular location because the location is close to their homes, for example, hotel and park. The correlation between locations also affects the location based social networks because some places are close to each other.

#### 5.1.3. Sequential Ordering

The users' chronological ordering is used to mention the subsequent visits of two locations. For example, we consider the two users with their location visiting pattern shown in Figure 4(c). For each visit of users, we can create an ordering of their similar location and preferences.

### 5.2. Challenges to Recommendations in LBSNs

The new LBSNs have three unique properties of locations. The unique properties of LBSN are location context awareness, the heterogeneous domain, and the rate of growth.

#### 5.2.1. Location Context Awareness

What kind of recommendation system is needed for LBSNs to consider the users current location, users location history, and the influences of location histories to other users?

#### 5.2.2. The Current Location of a User

Due to the following reasons, the current locations of users are more important parameter for generating recommendation system for LBSNs. The location granularity for different levels is represented by the current location of users. In recommendation system, it is very difficult to choose a proper granularity. If we choose hotel location of users that has a fine granularity, then a relative coarse granularity represents the town location of users.

The most visited location is near to the users compared to the location at far distance; this implies the distance property of locations. But also the quality of location is important for making recommendation system for LBSNs because of the ranking of recommendation system based on both the quality of locations and the location close to users. Another challenge is with respect to the collection of users' fine grain location, as it is frequently updated using mobile. By using efficient algorithms, the problem can be addressed with utilization of LBSNs. The future travel plan of users affects the current location due to location sequential property. For example, more numbers of people visiting some important place subsequently travel to the town.

#### 5.2.3. The Historical Locations of the User

The users' preference is indicated by the powerful histories of users' behaviors [104]. The LBSNs mention the user's historical location and also reflect the user's preferences, experiences, and living patterns compared to the online behaviors of users. It is not easy to model a location history of users because the location history depends on distance, hierarchy, and sequential properties of users. Based on the location history of users we have to learn user's personal preferences. Due to the following reason, it is very challenging work in LBSNs. (1) The challenging work is that we create users preference from sparse location data because a full set location history of users does not exist. (2) The user's location preferences are not only limited to their hotel and shopping locations because user has multiple kinds of interests: cycling, sports, movies, arts, and so forth. (3) Users preferences have granularity and also follow some hierarchical steps like snakes → food → pizza. (4) The user's preferences always depend on their location.

#### 5.2.4. The Location Histories of Other Users

Social opinion is one of the most important information bases for recommended system making up with location history generated by other users. From the location history we extract social opinions; it is not easy one because we are faced with the following challenges. (1) The continuous representation of user's changing location history is a complex task.  (2) For each location, user has different knowledge. For example, local user has expert knowledge to find high quality of hotel and shopping malls. It is easy to interface user's experiences and knowledge to the social opinion. From this users preference, we created a massive users location data. But for all locations, the same users do not have this much knowledge and location data.

#### 5.2.5. Heterogeneous Domain

The LBSNs heterogeneous graphs consisting of user and location node and three edges are user-user, location-location, and user-location edges. The LBSN is a model by at least three tightly associated graphs. The trajectories of LBSN can be eliminated by another node of social network. A location is an important object in the LBSN and also it consists of users information. The connection between two users is inferring heterogeneous graph of two objects. The similarity between two users is obtained by their location information sharing and will increase the strength of connection between the two users.

#### 5.2.6. The Rate of Growth

The growth of location based social networks is faster than normal social networks. LBSNs have higher links, nodes, and social structure compared with the normal one. The social network for academic is consisting of heterogeneous structure and conference with authors and papers. For this social network, adding new links is more difficult than LBSNs. Adding location to the LBSNs is easier than publishing a paper in academic social network. The rate of growth of LBSNs rises with high efficiency and standard scalability and updating new location to the recommender system. So, the research needs a year of experience regarding the users in LBSNs.

**Figure 5:**
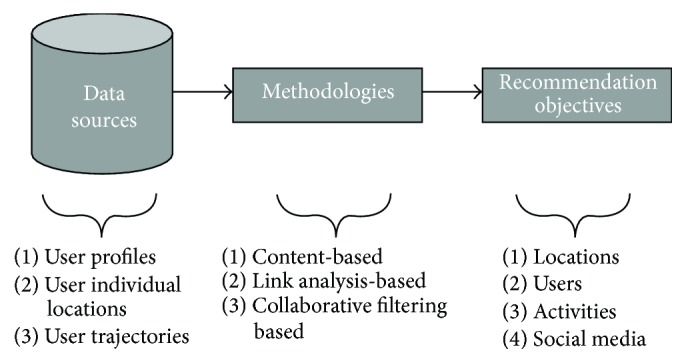
Categories of LBSN recommendation system, an overview.

Figure 5 shows the different types of social networks, such as social network for academic, social networks for general online, and location based social networks.

## 6. Proposed Location Recommendation System Using Social Pertinent Trust Walker Algorithm

In this section, we propose a social pertinent trust walker algorithm for an efficient location recommendation. In the proposed recommendation model, locations that are recommended to the user were predicted from the location based social network. Social pertinent trust walker algorithm determines the rating score of the locations based on the existing score rated for the similar location categories. After computing the rating score for the location categories, the list of locations with more relevance is recommended to the user.

**Figure 6:**
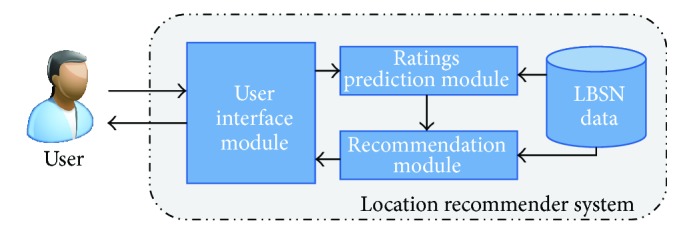
Proposed SPTW based location recommender system.

Social pertinent trust walker algorithm was introduced to reveal the more relevant suggestions and make recommendations more useful. The proposed recommendation model is portrayed in Figure 6. The proposed location recommender system comprises four major components, namely, user interface module, ratings prediction module, recommendation module, and location based social network.

**Figure 7:**
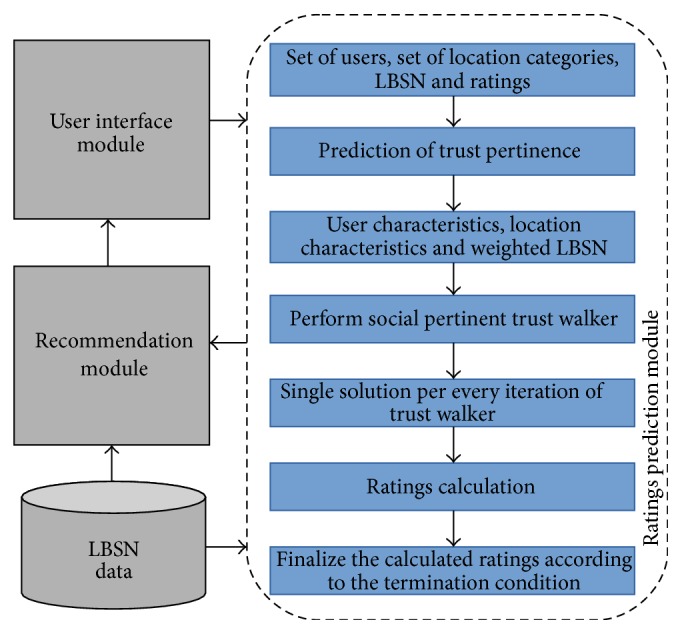
Overview of the proposed SPTW based location recommender system.

The proposed model can be restructured as in Figure 7. In the proposed location recommender system, the user's interaction is done through the user interface module. Then, the user requests are forwarded to the ratings prediction module. Here, the ratings for the categories of locations were predicted through proposed social pertinent trust walker algorithm. The proposed algorithm is the extended version of random walk proposed by Jamali and Ester [105]. To predict the ratings for the location categories, location based social network data is used. Then, the calculated ratings are forwarded to the recommendation module, where the ratings are exploited to make list of places as recommendations along with the help of LBSN data.

**Figure 8:**
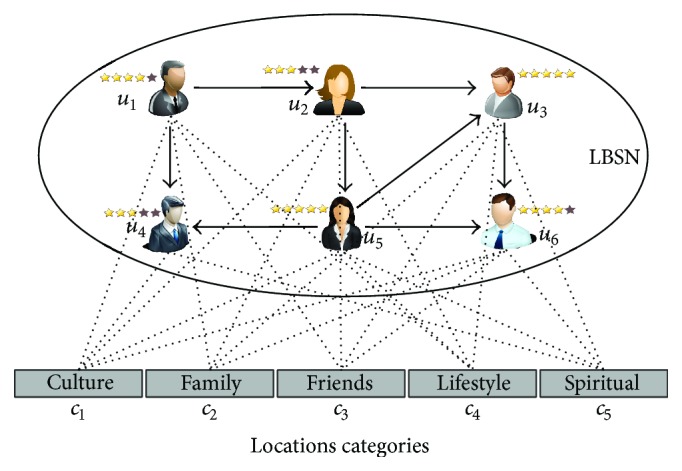
Location recommendations based on trust enhancement in LBSN.

The main ingredient of this work is trust between the users of a location based social network.  Figure 8 is representation of location recommendations based on trust enhancement in LBSN. Every user is capable of invoking with different categories of locations and rates the location categories based on their experiences. In a situation, while a user is requesting a location recommendation, the ratings for the particular user are calculated and the locations with the higher predicted ratings are recommended to the user. Therefore, utilization of LBSN data with ratings enhances the accuracy of recommendations.

Generally, the user set *U* = {*u*_1_, *u*_2_, *u*_3_,…, *u*_*k*_} and location categories set *C* = {*c*_1_, *c*_2_, *c*_3_,…, *c*_*n*_} are the major components of a trust based location recommender system. The user rating is expressed through a matrix called rating matrix and it can be denoted by *R* = [*R*_*u*,*c*_]_*k*×*n*_. The ratings can be any integers within a range. Mostly, the range of the ratings is between 1 and 5. One denotes poorly liked and five denotes heavily liked. In a location based social network, the trust between two users is denoted by real numbers 0, 1. The representation of trust is that one means full trust and zero means no trust. The trust between users is expressed through a trust matrix. In the trust matrix *T* = [*T*_*u*,*v*_]_*k*×*k*_, the relationship between the users or the trust between the users is denoted by nonzero value. Finally, the problem of location category recommendation can be defined as follows: user *u* requests a category of location *c* for which ratings are unidentified, hence the prediction of ratings *r* for user *u* though utilizing the ratings matrix *R* and trust matrix *T*.

### 6.1. Trust Relevancy

It is very well known fact that the trust relationship between the users of LBSN will not directly help in the enhancement of accuracy of recommendations. But the categories of locations recommended by the trusted users can be considered to be more reliable. Through this contradiction, the recommendation of location categories may be affected in the prediction of ratings for the particular user due to difference in preferences, interests, and observation. Hence, considering both similarities between the users and trust relationship may be more helpful to address the above challenge.

The trust relevancy between two users *x* and *y* can be defined as

(1) $$\begin{array}{c} \text{TruRel}=\text{SimUser}\left(\;x,y\;\right)*\text{Trust}\left(\;x,y\;\right), \end{array}$$

where SimUser(*x*, *y*) is the similarity between users *x* and *y* and Trust(*x*, *y*) is the degree of trust of user *x* to user *y*. The degree of trust between the users is calculated using the existing algorithms based on the historical interactions of the users [106]. Through calculating the trust relevancy between the connected users of location based social network, the weighted LBSN can be obtained. In order to calculate the similarities between the users, matrix factorization is used. To define matrix factorization, the user-location category rating matrix *R* can be decomposed into matrices, namely, *E* and *F*,

(2) $$\begin{array}{c} R\approx EF^{T}. \end{array}$$

The similarity between two users *x* and *y* can be calculated as follows:

(3) $$\begin{array}{c} \text{SimUser}\left(\;x,y\;\right)=\cos\left(\;x,y\;\right)=\frac{x\cdot y}{\left|x\right|\cdot \left|y\right|}. \end{array}$$

### 6.2. Social Pertinent Trust Walker Algorithm

In the proposed work, the SPTW algorithm has been designed to discover the interesting category of locations for the particular user from the location based social network. The SPTW algorithm reaches final solution after several iterations. For every iteration, the random walk initiates from the target user *Ux* in a weighted LBSN. While reaching the *n*th step of random walk in a trusted LBSN, the *user* is reached by the process. If *user* has rated the location category to be recommended, *CSx*, then this rating is used as *ratex* for that iteration. Else the random walk will be terminated based on the *Random*_*Probability*_*of*_*TrustWalk*_*user*,*CS*,*n*_. On the other hand, the trust walker continues its walk based on 1-*Random*_*Probability*_*of*_*TrustWalk*_*user*,*CS*,*n*_, one when the target user trusts the next node of weighted LBSN.

Algorithm 1 is a trust walk model for calculating rating for category of location for the specific user and it is evaluated using real time location based social network dataset (foursquare). The results prove that the algorithm is optimal and shows better performance through the evaluation metrics such as coverage, precision, and *F*-measure. Compared to the existing trust walk models, the proposed algorithm shows betterment in accuracy of recommendations. Based on the recommended category of locations from the proposed social pertinent trust walker algorithm, the list of top *k* nearest locations will be suggested to the user.

**Figure 9:**
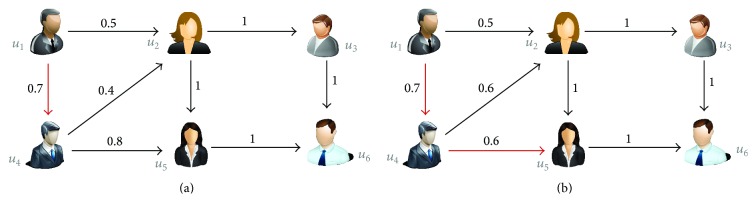
(a) Working of social pertinent trust walker (Step  1). (b) Working of social pertinent trust walker (Step  2).

The working of the SPTW algorithm is demonstrated in Figures 9(a) and 9(b). The edge between the users holds the probability as a weight. Let us consider location category *c*_4_which is considered to be recommended to the user *u*_1_. In the first step of trust walk as shown in Figure 9(a), the next node/target node is chosen as *u*_4_ based on larger probability edge value. If user *u*_4_ has rated the location category *c*_4_ with the rating* ratex*, the result of the trust walk will return as* ratex*. If the termination condition has not been reached, the iteration continues for the second walk. In this step, user *u*_5_ is chosen as specified in Figure 9(b). Again the algorithm checks whether the user *u*_5_ rated the location category *c*_4_. If the user *u*_5_ has not rated the location category *c*_4_, but the termination condition has been achieved, then the algorithm chooses the most similar location category from the list of location categories the user *u*_5_ has rated. The probability of selecting the similar service is based on *Rating*_*probability*_*user*_(*CSx*_*i*_). The rating of the chosen similar location category rated by the user *u*_5_ will be assigned as* ratex* and will be returned as a result for the iteration.

## 7. Group Recommendation

The proposed SPTW recommendation model is extended to group recommendation for the members of the particular group. The popularity of the POI plays a main role in the group recommendations. The proposed group recommendation model suggests POI to the group members who appeal for the travel recommendation. The proposed model is on the basis of the location category of the particular POI and the popularity of POI is used to find relevant location for the group.

**Figure 10:**
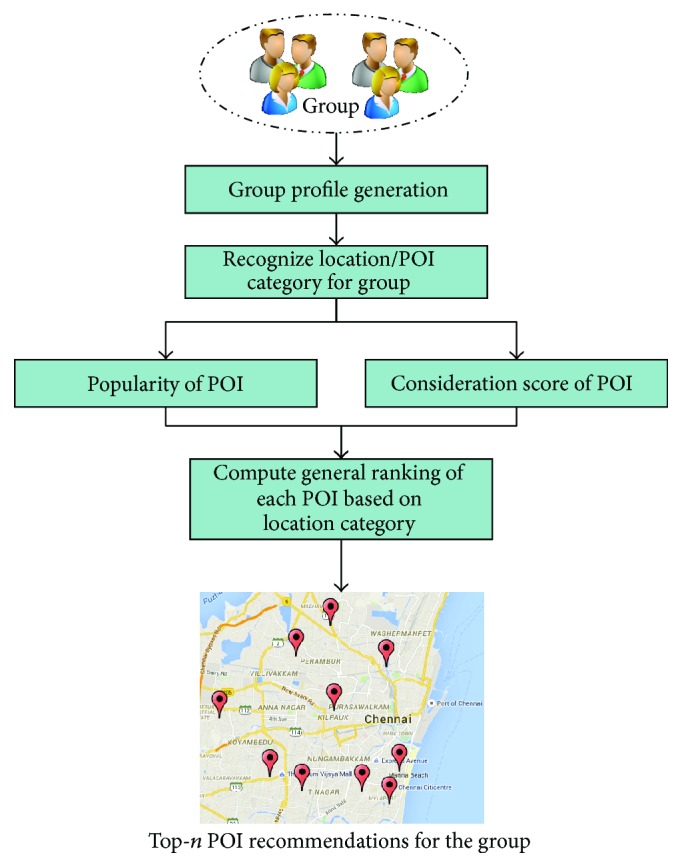
Proposed SPTW based group recommendation model.

In general, group members may be homogeneous or heterogeneous based on user preference regarding the locations. SPTW based group recommendation model (SPTW-GRM) exploits the interests of every user of the group along with the interested location category from their social profile. Interested location category with its repetition in the user profiles of the group members is used to form a group profile. The created group profile replicates the common interested location category with higher rating of the user's group. Later, based on location category relationship attributes, the proposed recommendation model determines POIs of the datasets whose exact category is not yet assigned. Then, the general ranking of the POIs is computed through proposed collective ranking function for the particular group. The computed general ranking of POI is based on the popularity of POI and the consideration score of POI for the group. The popularity of the POI represents the opinion of all users and their feedback regarding the particular POI. The consideration score of the POI reflects the repetition of the location category presented in the group profile. The process and working of the SPTW based group recommendation model are illustrated in Figure 10.

### 7.1. Group Profile Generation

The main aim of the proposed SPTW based group recommender system is to generate list of POIs to the group of users through analyzing the preference of every member of the group. The system creates a group profile that combines all users' preferences together to form group preferences. The group profile creation is adapted from an aggregation model of merging individual users into a group [107, 108]. The group reflects the combinational interests of group members with respect to location categories.

**Figure 11:**
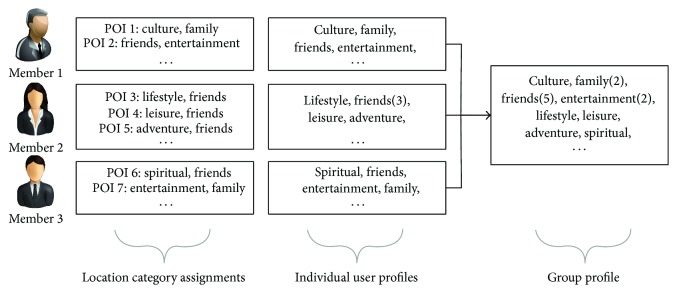
Group profile creation for the members of the group based on the location category of POI.

The proposed model finds the interesting location category for the particular user of the group to assign relevant location category to the user. Personal location category is classified by the user and it represents the user's interests towards POIs. Then, SPTW-GRM creates a common group profile to the members of the group and the profile includes the common location category assigned by the group members in their individual profiles. The location category with the higher repetition in the group profile shows the users' interests towards that location category and it has more impact on POI recommendations compared to the location categories with lower repetition.  Figure 11 shows the creation of group profile by SPTW-GRM considering the location category available on the individual user profiles of group members.

### 7.2. Location Category Relationship Attributes

SPTW-GRM depends on the location category relationship attributes to calculate the similarity between two location categories. The similarity computation process helps to identify more relevant POIs that should be considered to be part of top-*n* list. The location category relationship attributes are also used to determine the consideration score for the POI concerning particular group. The location category relationship attributes are calculated extensively by the similarity calculation process of the proposed SPTW algorithm. SPTW-GRM has adopted the similarity calculation model used by the SPTW algorithm and it classifies the location/POI based on its features.

### 7.3. Discovering Relevant POIs to Be Recommended

The number of POIs available on LBSN is high and to find a relevant POI in the vast list is a complex task. Analyzing each POI with respect to interests of group members is not an efficient way to generate recommendations. To reduce the number of comparisons, SPTW-GRM follows a specific filtering mechanism to create a set of location categories that has to be considered for POI recommendation. The specific filtering mechanism of the SPTW-GRM considers the personal location categories of the group members and includes the most similar location category with the list. Since the location category describes the POI, the location category can reflect the interest of the group too. The recommended POIs are the outcome of group preferences on location category used by the POI.

### 7.4. Group Recommendation Generation

After discovering relevant set of POIs based on location category, which is to be recommended to the group of users, SPTW-GRM proceeds to compute ranking for the POI based on popularity of the POI and consideration score for POI. Both features are combined together to form top-*n* POIs as recommendations. It is to be noted that top-*n* recommended POIs will have higher consideration scores with respect to the particular group taken into consideration.

#### 7.4.1. Popularity of POI

The popularity of the POI is used to find the rating of it by global users. Through this consideration, the users' satisfaction levels are obtained through the WoM (Word of Mouth) and feedback systems. The communal interests on the POI are generally expressed by the users as ratings on LBSNs. The highly rated POIs on the LBSN have higher ranks when it is considered for the recommendation. Popular POIs attract more users and have more check-ins on LBSNs. The SPTW-GRM analyzes the check-ins and ratings of the POIs based on group users' interests and location category considered. If the popularity of the POI is alone considered for the recommendations, then the relevance degree of the recommendations may be low and earlier research shows that such recommendations are useless. To enhance the accuracy of the POI recommendations, the popularity of the POIs should be considered along with the user's personalized needs. The popularity of the POI which is considered by SPTW-GRM is a decision factor to generate recommendations for all users who have check-ins at POIs of LBSNs.

#### 7.4.2. Consideration Score of POI

In parallel to the POI popularity computation, SPTW-GRM calculated the consideration score for POI with respect to the group. The degree of users' interest in a group for the POI is determined by SPTW-GRM by calculating the consideration score. The consideration score CScore(POI, *G*) collects the location category relationship attributes for all location categories in which group members are interested. The CScore of POI for group *G* is described as

(4) CScorePOI,G=∑lc∈G ∑poi∈POILLCRAlc,poi×RepetitionlcMax⁡RepetitionG×RepetitionpoiMax⁡RepetitionPOIL,

where LCRA(lc, poi) is location category relationship attributes of location category lc and point of interest (poi). The Repetition_lc_ is the repetition of location category in the group profile *G* and Max(Repetition_*G*_) denotes the maximum repetition in the group profile *G*. The Repetition_poi_ is the representation of repetition value of particular POI and Max(Repetition_POIL_) denotes the maximum repetition in the POI list. The weight of Repetition_lc_/Max(Repetition_*G*_), Repetition_poi_/Max(Repetition_POIL_) is used for location category and point of interest, respectively. Repetition_lc_ is the indicator that represents the user's interests on location category in the group. Larger value of Repetition_lc_ shows more importance of location category to the group. SPTW-GRM considers all these weighted attributes as consideration score while generating top-*n* POI as recommendation.

#### 7.4.3. General Rank Computation

After calculating the overall popularity and consideration score of each POI, SPTW-GRM estimates the general ranking for the POI through the CombMNZ, a linear combination measure proposed by Lee [109]. CombMNZ is used in many data fusion models and it takes multiple rakings of item *I* into consideration to calculate combined raking of item *I*. The CombMNZ model for POI can be represented as follows:

(5) $$\begin{array}{c} {\text{CombMNZ}}_{\text{POI}}=\overset{n}{\underset{\text{rl}=1}{\sum }}{\text{POI}}^{\text{rl}}\times \left|\;{\text{POI}}^{\text{rl}}>0\;\right|, \end{array}$$

where the number of ranked lists to be merged is represented by *n*. In other words, *n* is the number of ranked lists taken as inputs. The computed normalized score for all nonzero POI^rl^ of the POI for the ranked list rl is to be merged. Before calculating the general ranking score of the POI, the ratings of the individual rl should be converted into a range using the following equation:

(6) $$\begin{array}{c} {\text{POI}}^{\text{rl}}=\frac{{\text{Ratings}}^{\text{POI}}-{\text{POI}}_{min}^{\text{rl}}}{{\text{POI}}_{max}^{\text{rl}}-{\text{POI}}_{min}^{\text{rl}}}, \end{array}$$

where Ratings^POI^ is the ratings of the POI in the rl and POI_max⁡_^rl^ and POI_min⁡_^rl^ represent the maximum and minimum rating of POI in the rl.

The SPTE-GRM exploits the popularity of the POI and consideration score of the POI calculated in the earlier sections and it normalizes the value through the above equation. Then, the value of *n* is assigned to be 2 in CombMNZ calculation, since there are only two input sets, that is, popularity score and consideration score. The general ranking score of the POI is determined and the SPTW-GRM recommends top-*n* relevant POI to group members. Through utilization of data merger model, the proposed work explores the efficiency of consideration scores and popularity scores of the POI for efficient recommendations with higher popularity and relevance.

## 8. Experimental Evaluations

In this section, we show the results from the experiments to evaluate the performance of social pertinent trust walk algorithm through foursquare dataset. The proposed model is implemented in Java JDK 1.7 on Intel Core i7 3.1 GHz machine with 16 GB of memory running Microsoft Windows 7. Upcoming subsections describe the dataset and evaluation methodology with discussion of experimental results.

### 8.1. Dataset

Foursquare is a location based social network which holds user's previous visits to locations. Locations are termed as venues and the visits are called check-ins. Foursquare is a real dataset that consists of user ratings for venues and check-ins which is used to evaluate the quality of the recommendations made (Table 3). The foursquare data is used with our experimental setup with the extraction of user's social connections. This dataset contains 2153471 users, 1021970 check-ins, 1143092 venues, 2809581 ratings, and 27098490 trust relations which has been extracted from the foursquare application through the public API. As there is no classification of users based on gender in the dataset, we have adopted a classification model to classify the users anonymously into male and female for evaluation purpose. The possible user data from the dataset is classified into male users and female user sets.

The evaluation of social pertinent trust walker algorithm is done through comparing it with the following state-of-the-art methods. Golbeck [110] proposed a TidalTrust model which generates the ratings for the user through a trust inference algorithm. Massa and Avesani [111] have designed a model called MoleTrust, by which the trust score for the target user will be predicted through walking along social network. TrustWalker is a random walk model proposed by Jamali and Ester [105] which utilizes the similarity and trust. RelevantTrustWalker is an extension of TrustWalker introduced by Deng et al. [112] that uses the degree of trust between the users to predict the ratings.

The evaluation of SPTW-GRM for POI recommendation for group members is done on foursquare dataset. As the dataset is on individual users, we have created a modified dataset for the evaluation process. As there is no benchmark dataset available for POI recommendation for group users, we have adapted familiar approach to make group of users for evaluation process. The generation of user groups is done on basis of uniformity and size of the group. We have adapted group sizes from 2 to 8 to reach higher consensus between group members [113]. Based on similarity between users, the groups are classified into three types, namely, random, dissimilar, and highly similar groups. The similarity between users is calculated as

(7) $$\begin{array}{c} \text{Similar\_Users}\left(\;u,v\;\right)=\frac{\left|\left\{\text{poi}\;|\;\text{poi}\in {\text{POI}}_{u}\wedge \text{poi}\in {\text{POI}}_{v}\wedge \left|\text{rating}\left(u,\text{poi}\right)-\text{rating}\left(v,\text{poi}\right)\right|\geq 2\right\}\right|}{\left|\left\{\text{poi}\;|\;\text{poi}\in {\text{POI}}_{u}\vee \text{poi}\in {\text{POI}}_{v}\right\}\right|}, \end{array}$$

where POI_*u*_ represents the set of POIs rated by user *u* and poi is a point of interest and POI_*v*_ represents the set of POIs rated by user *v*. |rating(*u*, poi) − rating(*v*, poi)| ≥ 2 is the limitation of a poi shared by both users *u* and *v*. As the ratings are of traditional rating scale from 0 to 5, the similarity may be considered as high and low. Based on the similarity score computed by Similar_users(*u*, *v*), the users of the foursquare dataset have been grouped as highly similar and dissimilar groups with group size ranging from 2 to 8. We have also created random groups of users for the evaluation purpose. The random group may contain similar users or dissimilar users, but its size of users also ranges from 2 to 8. The groups are formed to hold uniform cohesiveness.

### 8.2. Evaluation Metrics

We adopt benchmark evaluation metrics to assess the performance and accuracy of the proposed SPTW recommendation method and the results were compared with the baseline recommendation algorithms. Generally, the accuracy of the recommendation algorithms is analyzed by root mean square error and we have also adopted RMSE to determine the error in generated recommendations

(8) $$\begin{array}{c} \text{RMSE}=\sqrt{\frac{\sum_{u,cs}{\left(R_{u,cs}-{\hat{R}}_{u,cs}\right)}^{2}}{N}}, \end{array}$$

where *R*_*u*,*cs*_ is the real rating user *u* has given to the category of location *cs* and ${\hat{R}}_{u,cs}$ is the predicted rating of user *u* to the category of location *cs*. The total number of tested ratings is denoted by *N*. The larger value of RMSE denotes the inaccuracy of the recommendation algorithm whereas the smaller value of RMSE represents the preciseness of the algorithms.

Due to the high sparsity of the dataset used, some recommendation algorithms are not capable of predicting the ratings. Utilizing the trust between the users increases the coverage and precision ratio. Coverage metric is used to calculate the predicted value of generated pair of the user and category of locations. In any circumstances, if the recommendation algorithm is not capable of predicting the similarity between the user and category of locations, then it is said that the algorithm did not cover the particular pair of user and category of location:

(9) $$\begin{array}{c} \text{Coverage}=\frac{CS}{N}, \end{array}$$

where *CS* denotes the number of ratings predicted and *N* denoted the number of ratings tested.

RMSE and coverage can be combined into single metric as *F*-measure. To calculate *F*-measure, it is necessary to determine precision values. Precision values can be obtained from RMSE. For such calculation, RMSE value should be converted into precision metric range of 0 to 1:

(10) $$\begin{array}{c} \text{Precision}=1-\frac{\text{RMSE}}{\text{Maximum Possible Error}}. \end{array}$$

In the above equation, RMSE is divided by the maximum possible error, that is, 4. Here, 4 is considered as maximum possible error due to the ratings in the range of 1 to 5.

*F*-measure is determined by using coverage and precision as follows:

(11) $$\begin{array}{c} F\text{-Measure}=\frac{2\times \text{precision}\times \text{coverage}}{\text{precision}+\text{coverage}}. \end{array}$$

NDCG is the normalized version of DCG (Discounted Cumulative Gain) which is defined as

(12) $$\begin{array}{c} \text{DCG}@k\left(\;u\;\right)=\overset{k}{\underset{j=1}{\sum }}\frac{g_{u,\text{poi}\left(j\right)}}{\max\left(1,\log_{b}j\right)}, \end{array}$$

where *g*_*u*,*i*_ is the gain of each user (*u*) when point of interest (poi) is recommended and *j* is the position of the poi in the recommendation:

(13) $$\begin{array}{c} \text{NDCG}@k\left(\;u\;\right)=\frac{\text{DCG}@k\left(u\right)}{{\text{DCG}}^{*}@k\left(u\right)}. \end{array}$$

Here, DCG^*∗*^ is the ideal DCG, where pois are arranged in the decreasing order till the position of *k* with respect to *R*_*u*,poi_.

For a list of *k* points of interest, an average NDCG is defined as follows:

(14) $$\begin{array}{c} \text{NDCG}@k=\frac{1}{u_{0}}\overset{u_{0}}{\underset{u=1}{\sum }}\text{NDCG}@k\left(\;u\;\right). \end{array}$$

MAE is the Mean Absolute Error used to evaluate the proposed model and it is defined as

(15) $$\begin{array}{c} \text{MAE}=\frac{\sum_{\left(u,poi\right)\in R_{\text{test}}}^{}\left|{\text{PR}}_{u,\text{poi}}-{\text{AR}}_{u,\text{poi}}\right|}{\left|R_{\text{test}}\right|}, \end{array}$$

where PR_*u*,poi_ denotes the predicted ratings for user *u* for poi and AR_*u*,poi_ represents the actual ratings and *R*_test_ is the set of users and point of interests used for test purpose.

### 8.3. Experimental Results

**Figure 12:**
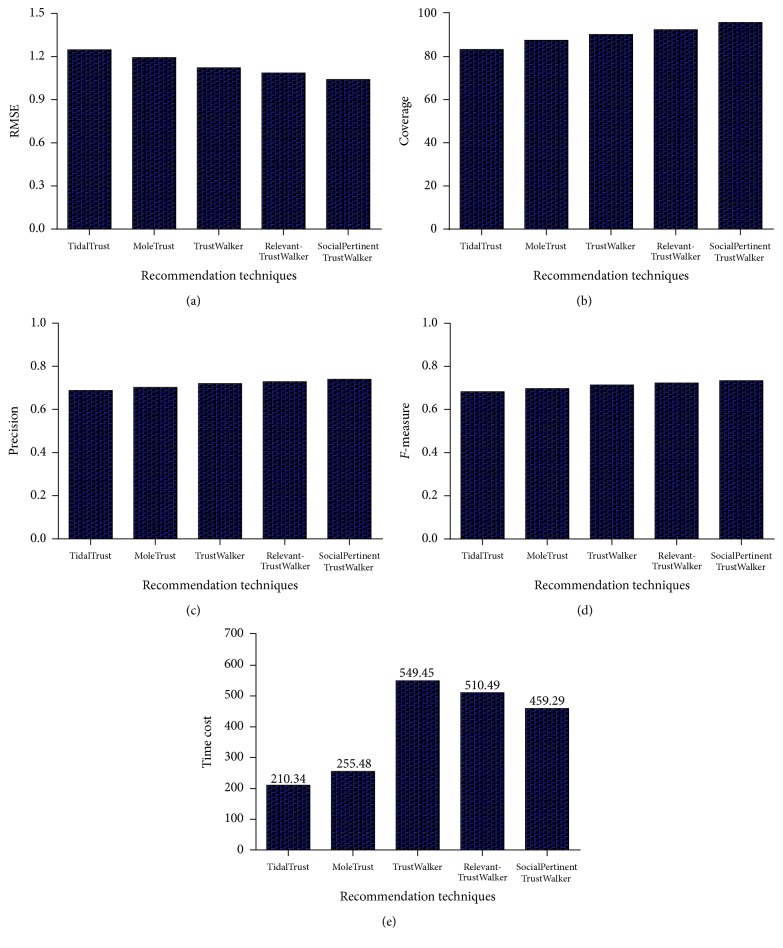
(a) Comparison of RMSE. (b) Comparison of coverage. (c) Comparison of precision. (d) Comparison of *F*-measure. (e) Comparison of time cost utilized by different algorithms.

The results of the various experimental comparisons with recommendation results and performance evaluation of different algorithms are presented in this subsection. From the experimental results of Table 4 and infographics of Figures 12(a), 12(b), 12(c), and 12(d), it is clear that there is no complete gain for trust based algorithms in terms of recommendations accuracy. Though other models consider trust of the users for recommendations, the proposed SPTW combines both trust of users and similarity between users along with the current location. By this, the accuracy of SPTW has been improved a lot compared to other methods and the performance of recommendation is better compared to other algorithms. Obviously, SPTW has less RMSE compared to other algorithms due to the selection of appropriate users based on trust and similarity and the coverage of SPTW is also high among other algorithms. While considering the metric of *F*-measure, SPTW performs very well along with other algorithms considered for comparison. Due to enormous size of dataset, the time taken for location recommendation should be also considered as an evaluation factor.  Figure 12(e) portrays the average time cost consumed by various algorithms taken for consideration. Since trust calculation is a bit additional task added to the prediction and recommendation process, it makes an impact over computational time cost. Compared to other models that consider trust between the users, SPTW costs less time because, in every iteration or walk, SPTW chooses target node based on probability and trust relevancy. Such an approach makes SPTW algorithm more reliable and helps to make recommendations more quickly through the improved computational efficiency.

**Figure 13:**
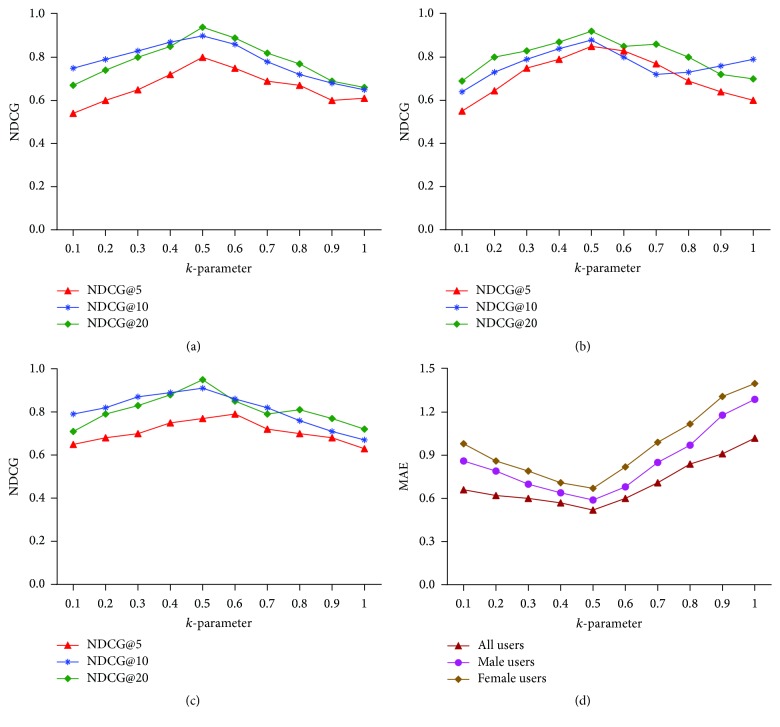
(a) Comparison of NDCG for all users. (b) Comparison of NDCG for male users. (c) Comparison of NDCG for female users. (d) Comparison of MAE for male, female, and all users.

The evaluation of the SPTW algorithm and its POI recommendation score were done as assessing a traditional information retrieval system. Hence, we have adapted famous evaluation metrics NDCG (Normalized Discounted Cumulative Gain) and MAE (Mean Absolute Error). NDCG is used to determine the performance of the proposed model through analyzing the relevant POIs in the recommendation list. MAE is used to find the number of errors in the generated recommendations. The large value of MAE shows the higher error with the recommendations. Figures 13(a), 13(b), and 13(c) portray the NDCG comparison of all, male, and female users, respectively. The tuning parameter *k* plays a key role in the performance of SPTW algorithm. The weights of the tuning parameter *k* are used to identify the optimal performance of SPTW algorithm in various datasets. The optimal performance is obtained in all users and male dataset when *k* = 0.5 for all NDCG@5, 10, and 20. In the female users dataset, the optimal performance is obtained when *k* = 0.6 for NDCG@5 and *k* = 0.5 for NDCG@10, 20. The comparison of MAE for male, female, and all users datasets is depicted in Figure 13(d). As the behavior of the users' choice selection randomly differs, it is also to be considered that the dataset is very sparse. The sparsity of the dataset can be addressed using the tuning parameter. The results of the MAE show that when the tuning parameter *k* = 0.5, SPTW shows optimistic results and it can be followed in the future experiments and evaluations.

**Figure 14:**
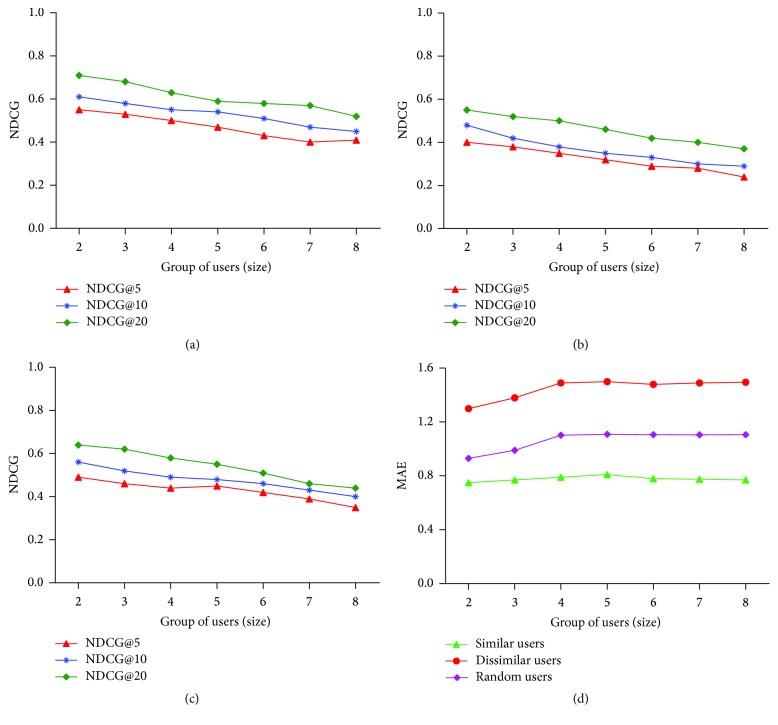
(a) Comparison of NDCG for highly similar users. (b) Comparison of NDCG for dissimilar users. (c) Comparison of NDCG for random users. (d) Comparison of MAE for highly similar, dissimilar, and random users.

**Figure 15:**
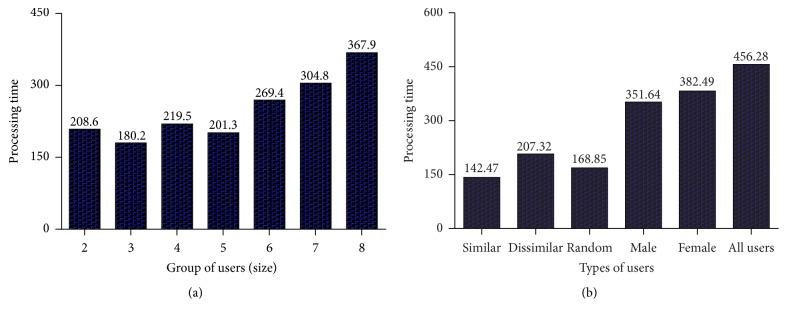
(a) Comparison of processing time of SPTW-GRM for various group sizes. (b) Comparison of average processing time of SPTW-GRM for different users.

The group recommendation based on SPTW-GRM is evaluated on the uniformly distributed group with the sizes ranging from 2 to 8. The performance of the SPTW-GRM is evaluated using NDCG and MAE for performance and efficiency. SPTW-GRM is implemented on the formed groups on foursquare dataset based on similarity between users as highly similar, random, and dissimilar groups. Popularity of POI and consideration score increase the accuracy of the groups. The complete comparison of NDCG is represented in Figures 14(a), 14(b), and 14(c) for highly similar, random, and dissimilar groups, respectively. The comparisons of MAE for highly similar, dissimilar, and random users are portrayed in Figure 14(d). The difference between the results of the SPTW-GRM for various groups with various users' sizes shows the statistical significance of the work. From the results, the observations are done to determine the uniformity of the group members in the various groups. The highly similar groups hold higher uniformity levels compared to other groups. The recommendations for this group are generated quickly compared to the other two types. The random group comes next in the performance and the dissimilar groups hold last position in the performance, efficiency, and accuracy of recommendations. As a next step in the evaluation of the SPTW-GRM, the efficiency of the model is analyzed for various group sizes regardless of its type. The processing time is computed for all group sizes and average is taken. The average processing time increases as the size of the group increases. The trend of average processing time denotes the scalability of the SPTW-GRM and Figure 15(a) represents the average processing time of group users. We have also evaluated all types of user sets to determine the efficiency through average processing time. The comparison of average processing time of SPTW-GRM for different types of user sets is portrayed in Figure 15(b). The average processing time shows the time taken for recommendation generation depending on group size and uniform levels between the users. The results of the SPTW-GRM show that one user can influence another user's opinion. In other words, satisfaction levels of one user can have an impact on another user of the group through the generated recommendation.

## 9. Conclusion

This final section is the summary of the work presented in this paper, which describes the key points that should be taken into consideration by the researcher, who is aiming to develop a recommender system. This paper creates an impact through the outline of several future work challenges in the area of recommender systems' design and development. Through the analysis of interfaces used by the recommender systems, it is very well noticed that the recent development of mobile platforms has been utilized very little. Clever exploitation of mobile platform with the personal data such as current location may help in providing precise recommendations to users in an improved manner. Most of travel recommender systems lack the points of personalization, interactivity, and adaptivity. Though TRS provide points of interests as their suggestion according to the user preferences, the system still needs user's help to build their trip manually. Some research has tried to solve the issue of automaticity in travel planning service, but still the problem of automatic travel planning is yet to be addressed. This is a novel issue where social information and context of the user can be utilized to solve the problem.

Every classical approach (such as collaborative, content-based, and demographic) suffers from various problems in providing personalized recommendations to the individual user. The trend of hybrid recommendation models along with the contextual information of the user may solve such issues of individuality. Usage of the implicit and explicit preferences of users extended with the semantic models addresses the problem of uncertainty in the recommendation process. An artificial intelligence technique such as knowledge representation is commonly used for reasoning the recommendation process. Automatic clustering algorithms may be used to classify users and optimization techniques can be deployed to generate the cost effective recommendations to the user. The problem of complexity in the scheduling and planning process of route generation is a novel problem in the travel route recommendation. The best way of obtaining more information from the user is the exploitation of their social network data. Since tourism domain is very social in nature, this data may help much in the development of recommender systems based on user interests. Utilization of the social data such as check-in behavior, ratings, social relationships, and recent area of work can help in the discovery of more accurate travel recommendations, which fits better the tastes of the user.

For every recommender system, it is very important to hold specific information about users and their interests as a profile. The development of new learning mechanisms to analyze interactions of a user with the system and its ability to convert it into user preference can make recommender system more dynamic in providing suggestions. As a hybrid approach utilization of ontologies may be used to represent the user's preferences in the semantic manner, such approach can overcome difficulties in the lack of personalization with the textual information. The location information is already used by many recommender systems, which can be followed by utilization of device sensors' data such as RFID signals, weather temperature, and health metrics/signals. Initially, recommender systems were focusing on filtering mechanisms to improve the accuracy of recommendations. Now, hybrid algorithms incorporated with the various factors-influenced data have been taken into consideration in the development of efficient recommendation models.

The rapid growth of social media sites created a wide opportunity to build social recommender systems. The clustering of users, according to their tastes as a similar metric, can generate good recommendation in more efficient manner. Investigation of problems such as influence of friends and their distance, time-series information, acquisition of tastes of individual user, enabling of privacy and security of the users and their data, dynamic variations and places of the user, automated analysis of heterogeneous data through a flexible framework, practical situation influenced improvement of performance, and cold start problem may be considered in the discovery and development of new hybrid recommender systems. As a crucial conclusion, the success of recommender systems purely depends on the effective learning of user behavior and generation of user acceptable recommendations.

Location recommendation system is proposed and evaluated using foursquare dataset. The ratings are predicted through social pertinent trust walker algorithm. The results are compared with the other existing TrustWalker methods, namely, TidalTrust, MoleTrust, TrustWalker, and RelevantTrustWalker. It is observed from the experimental results that RMSE, coverage, precision, and *F*-measure for the proposed SPTW based location recommender system achieve the best performance. Compared to other models that consider trust between the users, the SPTW costs less time because, in every iteration or walk, SPTW chooses target node based on probability and trust relevancy. Such an approach makes the SPTW algorithm more reliable and helps to make recommendations more quickly through the improved computational efficiency.

The SPTW based location recommendation system is extended for the group of users. The system is evaluated for group of users recommendations through the groups formed in foursquare dataset. The group size ranges from 2 to 8 and the groups are classified as highly similar, random, and dissimilar. The groups are classified on the basis of uniformity between users to maintain cohesiveness and bondage. The results are evaluated based on NDCG and MAE metrics. The inference on the results of evaluation process shows that the efficiency and performance of the model reduce as the size of the group increases. The ultimate outcome of the results shows that the groups with more similar users perform well compared to random users and dissimilar users. The average processing time taken for the various types of groups also supports the performance inference of the recommendation results. SPTW-GRM proves its efficiency and performance on both recommendations for individual users and group of users.

### 9.1. Future Work Guidelines

In this section, we quickly remark the important issues that are examined at present in the advancement of recommender system frameworks in e-Tourism domain, such as

- broadening of the recommendations offered to the client;
- utilization of social information accessible in the present Web 2.0 apps;
- change in improved recommendations through utilizing the additional capacities of smart mobile phones.

*What Is the Need for Content-Based Systems in Tourism?* The main concentration of the content-based systems is to recommend things to the users based on their profile, by generating the user specific results which may fascinate the user. In the area of tourism, this may be considered as an essential issue of travel recommender systems. Few recommender systems try to promote new spots or new activities, which makes the recommendations ineffective. A good travel recommender system provides broad suggestions to the users and allows them to choose their routes with activities. The list of user's attractions should be synchronized with rating limit in order to maintain the quality of recommendations. The usage of multiple techniques to filter the activities for the recommendation generation is a new scope in this domain. The clustering of items into groups with similar attributes is an effective mechanism in the recommendation process to build a good list of suggestions by utilizing every cluster of items for a user specific proposition of their tourist interests.

*Is Growth of Web Treated as an Issue with Travel Recommender Systems?* In the present scenario, recommender systems use the strength of web based applications with their social network exposure. Through offering social tools, web apps promote the use of filtering mechanisms such as collaborative filtering. It is well known that these collaborative filtering techniques allow rating of items and collecting data of the user in the social level or individual level. The systems which use such mechanisms are called social recommender systems. These available social tools can be used to differentiate the cluster of users from cluster of items. In an existing work, the users hold a related tag cloud pertinent to their profile and another tag is created for their interests. Such a kind of data is used to discover the coincidence between the users and items. Some travel recommenders grab the data regarding the connections of the users along with the analysis of searches and readings in the wiki. These recommender systems utilize this data to calculate the satisfaction degree of the user for the particular article. Another existing recommender system keeps up the social media profile of the user to consider the contact information and to examine the communication between other users. The main concept while considering the social recommender systems is trust. Trust is very important challenge as it deals with the ratings of the items and the reliability of the user. While addressing the issues in the segment of trust in social recommender systems, the significant aspect to be considered is that the user with higher reliability score should be treated with higher weights compared to others with the lower weights.

*Why Adaptability Is Considered as Very Important Quality of Travel Recommender Systems?* A unique feature of tourism domain is the area where the recommenders have been used, as it adapts to the users and helps them through generated suggestions in different places and in different moments. These travel recommender systems have begun to fuse context aware mechanisms with it. The accomplishment of this methodology is because of the far reaching utilization of Smartphones. Numerous tourism recommenders keep running mobile devices, so the location of the user may be utilized in the filtering process of items to be demonstrated. In contrast to the existing systems, the enhancement of location consideration has to be modified. The present location of the user is important. But along with it, the significance of other spots visited already should also be analyzed and used in the recommendations. In tourism recommender systems, different highlights are considered as related data for an event. For example, the present climate is analyzed to choose appropriate outdoor or indoor activities to be recommended.

*What Is the Requirement of Hybrid Recommendation Models in TRS?* In a travel recommendation framework, a multifarious model of the context is considered for building customized route arrangements. The extracted context data is sorted out in an order, with features identified with the climate, travel traffic, security (such as parking, mobile connectivity, and medicinal facilities), service facilities (vehicle service station, etc.), and vacation spots (beach, entertainment place, fishing zone, etc.). The generally defined four prime parameters of context are location (e.g., current location of the of the user and the locality of spot), time (time required by the client to achieve the spot, the opening/shutting times, etc.), climate and natural conditions (e.g., temperature, mugginess, precipitation degree, wind, season, snippet of the day), and social elements (number of clients near to the spot and number of positive/negative inputs). These aspects were formulated as key points in the accomplishment of travel recommender systems in the area of e-Tourism, due to the intrinsic mobile activity of the users in this particular application area.

## Figures and Tables

**Algorithm 1:**
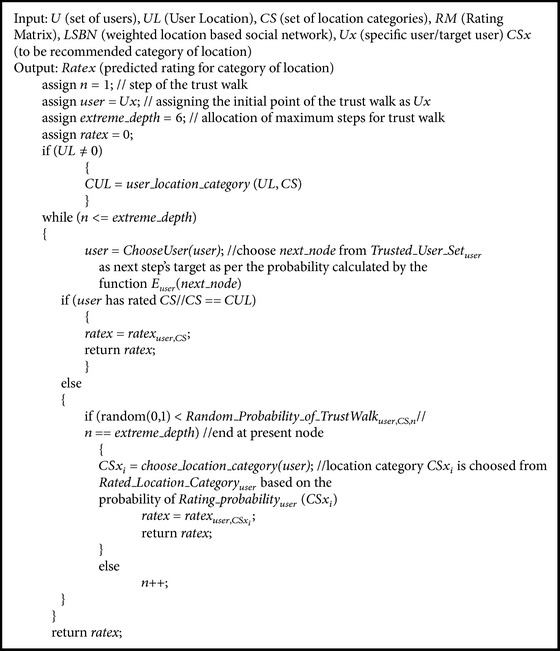


**Table 1:** Comparison of interface and functionality of travel recommender systems.

Articles	Interface			Functionality				Context aware system
	Web	Mobile	Hybrid	Attractions suggestion	Destination recommendation/tourist package	Trip planner	Social features	
Jannach et al. [48]	**✓**			**✓**				
Mínguez et al. [62]	**✓**					**✓**		
Ricci et al. [52]		**✓**		**✓**				
Sebastia et al. [63]	**✓**			**✓**		**✓**		
Vansteenwegen et al. [44]	**✓**	**✓**	**✓**	**✓**		**✓**	**✓**	**✓**
Borràs et al. [64]	**✓**			**✓**				
Garcia et al. [65]	**✓**			**✓**		**✓**	**✓**	
Gavalas and Kenteris [51]	**✓**	**✓**	**✓**	**✓**				**✓**
Lorenzi et al. [59]	**✓**			**✓**	**✓**	**✓**		
Luberg et al. [66]	**✓**			**✓**		**✓**		
Martin et al. [55]		**✓**		**✓**				**✓**
Montejo-Ráez et al. [46]	**✓**			**✓**		**✓**		
Rey-López et al. [54]	**✓**	**✓**	**✓**			**✓**	**✓**	**✓**
Wang et al. [49]	**✓**			**✓**		**✓**		
Batet et al. [67]		**✓**		**✓**		**✓**		**✓**
Borràs et al. [68]	**✓**	**✓**	**✓**	**✓**		**✓**		**✓**
Koceski and Petrevska [61]	**✓**			**✓**	**✓**			
Martínez-Santiago et al. [53]		**✓**		**✓**				**✓**
Noguera et al. [58]		**✓**						**✓**
Braunhofer et al. [56]		**✓**		**✓**				**✓**
Garcia et al. [69]		**✓**		**✓**				**✓**
Gyorodi et al. [70]	**✓**			**✓**				
Kurata and Hara [71]	**✓**			**✓**		**✓**		
Lucas et al. [72]	**✓**			**✓**		**✓**		
Meehan et al. [73]		**✓**		**✓**			**✓**	**✓**
Ruotsalo et al. [74]	**✓**	**✓**	**✓**	**✓**				**✓**
Savir et al. [75]	**✓**			**✓**		**✓**		
Umanets et al. [57]	**✓**	**✓**		**✓**			**✓**	**✓**
Yang and Hwang [76]		**✓**		**✓**			**✓**	**✓**

**Table 2:** Comparison of AI techniques used by travel recommender systems.

Articles	Multiagent system	Optimization technique	Automatic clustering	Management of uncertainty	Knowledge representation
García-Manotas et al. [84]		*✓*			
Sebastia et al. [63]	*✓*				*✓*
Vansteenwegen et al. [44]		*✓*			
Fenza et al. [85]			*✓*		
Garcia et al. [65]					*✓*
Gavalas and Kenteris [51]			*✓*		
Lorenzi et al. [59]	*✓*				
Wang et al. [49]				*✓*	*✓*
Batet et al. [67]			*✓*		*✓*
Hsu et al. [86]				*✓*	
Martínez-Santiago et al. [53]					*✓*
Noguera et al. [58]			*✓*		
Garcia et al. [69]		*✓*			
Lucas et al. [72]			*✓*	*✓*	*✓*
Meehan et al. [73]		*✓*		*✓*	
Moreno et al. [87]			*✓*		*✓*
Ruotsalo et al. [74]			*✓*	*✓*	*✓*

**Table 3:** Foursquare dataset statistics.

Features	Gross units
Users	2153471
Venues	1143092
Check-ins	1021970
Ratings	2809581
Trust relations	27098490

**Table 4:** Comparison and performance evaluation.

Algorithms	RMSE	Coverage (%)	Precision	*F*-measure	Time cost
TidalTrust	1.247	83.25	0.6883	0.6826	210.34
MoleTrust	1.193	87.58	0.7018	0.6967	255.48
TrustWalker	1.122	90.23	0.7195	0.7138	549.45
RelevantTrustWalker	1.086	92.47	0.7285	0.7228	510.49
SocialPertinent TrustWalker	1.041	95.78	0.7398	0.7341	459.29
